# Hepatocyte Permissiveness to *Plasmodium* Infection Is Conveyed by a Short and Structurally Conserved Region of the CD81 Large Extracellular Domain

**DOI:** 10.1371/journal.ppat.1000010

**Published:** 2008-02-29

**Authors:** Samir Yalaoui, Sergine Zougbédé, Stéphanie Charrin, Olivier Silvie, Cécile Arduise, Khemais Farhati, Claude Boucheix, Dominique Mazier, Eric Rubinstein, Patrick Froissard

**Affiliations:** 1 Université Pierre et Marie Curie-Paris6, UMR S511, Paris, France; 2 Inserm, U511, Paris, France; 3 Inserm, U602, Villejuif, France; 4 Université Paris 11, Institut André Lwoff, Villejuif, France; 5 AP-HP, Groupe Hospitalier Pitié-Salpêtrière, Service Parasitologie-Mycologie, Paris, France; Instituto Gulbenkian de Ciência, Portugal

## Abstract

Invasion of hepatocytes by *Plasmodium* sporozoites is a prerequisite for establishment of a malaria infection, and thus represents an attractive target for anti-malarial interventions. Still, the molecular mechanisms underlying sporozoite invasion are largely unknown. We have previously reported that the tetraspanin CD81, a known receptor for the hepatitis C virus (HCV), is required on hepatocytes for infection by sporozoites of several *Plasmodium* species. Here we have characterized CD81 molecular determinants required for infection of hepatocytic cells by *P. yoelii* sporozoites. Using CD9/CD81 chimeras, we have identified in CD81 a 21 amino acid stretch located in a domain structurally conserved in the large extracellular loop of tetraspanins, which is sufficient in an otherwise CD9 background to confer susceptibility to *P. yoelii* infection. By site-directed mutagenesis, we have demonstrated the key role of a solvent-exposed region around residue D137 within this domain. A mAb that requires this region for optimal binding did not block infection, in contrast to other CD81 mAbs. This study has uncovered a new functionally important region of CD81, independent of HCV E2 envelope protein binding domain, and further suggests that CD81 may not interact directly with a parasite ligand during *Plasmodium* infection, but instead may regulate the function of a yet unknown partner protein.

## Author Summary

Minutes after the bite of a female mosquito, the malaria parasite *Plasmodium* enters the liver where it invades liver-specific cells called hepatocytes and undergoes one round of multiplication. This stage is a prerequisite to the blood stages of the life cycle which cause the malaria symptoms. The invasion of hepatocytes probably requires a series of interaction between the host cell and the parasite, but the exact mechanisms are still elusive. CD81, a protein of the tetraspanin superfamily, is the only hepatocyte surface protein that has been shown to be strictly required for the infection by the malaria parasite. We have here studied the regions of CD81 that are important for infection, by exchanging segments with the corresponding parts of a closely related molecule, or by mutating discrete residues. This study has uncovered a new functionally important region of CD81 and, by comparing the ability of several CD81 antibodies to block infection, has strengthened the hypothesis that CD81 might regulate the function of another molecule present at the hepatocyte surface during *Plasmodium* infection. The region of CD81 identified here is different from the region involved in the binding of the hepatitis C virus.

## Introduction

Malaria remains the most important parasitic human disease, responsible for millions of deaths each year. *Plasmodium* infection is initiated by the inoculation of sporozoites in the host by a female *Anopheles* mosquito. Within minutes of biting, the motile sporozoites join the liver and infect hepatocytes, where they further differentiate into a replicative exo-erythrocytic form (EEF) that will ultimately give rise to thousands of merozoites that initiate the pathogenic erythrocytic cycle. Like other Apicomplexa parasites, *Plasmodium* invades host target cells actively, using a parasite actin-myosin motor machinery to translocate a junction formed between parasite ligands and host cell receptors. This moving junction results in the internalization of the parasite through an invagination of the host cell plasma membrane, resulting in the formation of the parasitophorous vacuole where the parasite further develops [1]–[4]. The nature of the molecular interactions mediating sporozoite invasion of hepatocyte still remains elusive. Two well-characterized sporozoite surface proteins, the circumsporozoite protein and the thrombospondin-related adhesive protein, are known to interact with the liver heparan sulphate proteoglycans [5]–[7] which are responsible for the initial sequestration of sporozoites in the liver sinusoids [8],[9]. More recently, two *P. berghei* proteins belonging to the *Plasmodium* 6-Cys domain protein family and specifically produced in liver-infective sporozoites, Pb36p and Pb36, were shown to be necessary for sporozoite infection [10]. On the hepatocyte side, the only surface protein known to play a key role in the infection by several *Plasmodium* species is the tetraspanin CD81. Indeed, antibodies to CD81 or CD81 silencing strongly reduce the infection of hepatocytic cells by *P. yoelii* (a rodent parasite) and *P. falciparum* (a human parasite) sporozoites. Additionally, *P. yoelii* sporozoites fail to infect CD81-deficient mouse hepatocytes both in vitro and in vivo [11]–[13]. Depending on the host target cell, another rodent parasite, *P. berghei*, also uses CD81 for sporozoite invasion [14].

CD81 belongs to a family of proteins called tetraspanins which have been implicated in various biological processes such as cell adhesion, migration, cell fusion, co-stimulation, signal transduction, and differentiation (reviewed in [15]–[17]). They play a role in the infection by several viruses including HIV and CD81 is essential for the infection of hepatocytic cells by the hepatitis C virus (HCV) [18],[19]. CD81 presumably acts as a receptor for HCV as it binds to HCV E2 envelope glycoprotein [18],[19].

The molecular function of tetraspanins remains uncertain [15]–[17]. Biochemical studies have shown that that tetraspanins associate with many other surface molecules [20], and they have been suggested to function as molecular organizers of membrane multi-molecular complexes, collectively referred to as the “tetraspanin web” [21]. Within this network of interactions, tetraspanins form primary complexes with a limited number of proteins called tetraspanin partners. These tetraspanin/partner interactions are direct and highly specific. For example, the tetraspanin CD151 associates directly with the integrins α6β1 and α3β1 and functionally regulates these integrins [22],[23]. CD9P-1 and EWI-2, two related Ig domains proteins are direct partners of both CD9 and CD81, including in hepatocytes [24]–[27]. These primary interactions resist to detergents such as digitonin (and in some cases Triton X-100), and typically occur at a high stoichiometry [22]–[27]. Tetraspanins also interact with one another, building specific proteo-lipidic membrane microdomains to which they probably target their partner proteins [15]–[17],[28],[29]. The localization of CD81 in these microdomains is likely to be important for the function of CD81 in sporozoite infection since modulation of cellular cholesterol levels, that changes tetraspanin microdomain organization, also modifies the extent of CD81-dependent sporozoite invasion [13],[29].

The molecular determinants of CD81 function in sporozoite infection are not known. Tetraspanins have 4 transmembrane domains that delimitate 3 short cytosolic regions and 2 extracellular domains of unequal size (the small extracellular loop (SEL) and the large extracellular loop (LEL)) [15]–[17]. The transmembrane domains are the most conserved and are believed to contribute to functions common to the different tetraspanins, possibly to the interaction with one another and with lipids. The crystal structure of CD81 LEL has been solved [30]. Two antiparallel α-helices (A and E) in the continuity of the third and fourth transmembrane domains form the stalk of the domain. The A helix is connected through a short loop with a 3^rd^ helix (B) and molecular modelling indicates that these 3 helices, although not conserved at the amino acid level, form a structure common to all tetraspanins [31]. Two additional helices (C and D), inserted in the stalk, contribute to form with the B helix the head subdomain of the LEL. Several conserved cysteines, located after the B helix (a CCG motif hallmark of tetraspanins), between the C and D helices and at the beginning of the E helix, contribute to the maintenance of the LEL structure. Other tetraspanins have also a C and D region, which are not structurally conserved [31]. The large extracellular domain has been shown to be implicated in specific tetraspanin functions. For example, the LEL of CD9, CD81 and CD151 contribute to the interaction with EWI-2, CD19 and integrins respectively [23],[25],[32]. In addition, the binding of the HCV envelope glycoprotein E2 to CD81 and the function of CD9 in sperm-egg fusion critically depend on a Phe residue present in the D region of the LEL [33],[34].

The aim of this study was to uncover the residues of CD81 that are critical for host cell infection by sporozoites. In a first phase of this study, we used several CD9/CD81 chimeras, based on the fact that although among tetraspanins CD9 is the closest to CD81, with 45% identity at the amino acid level, it does not support *Plasmodium* sporozoite invasion. The availability of the crystal structure made it possible to generate inter-domain (or subdomain) swaps with presumably minimal influence on the overall conformation of the chimeric molecules. In a second step, following an analysis of CD81 crystal structure, and previous modelling studies [30],[31], we identified several residues of a solvent-exposed region that are critical for infection. This is the first demonstration that residues of the LEL located outside the C and D helices play a critical role in a specific tetraspanin function. These findings have implications for understanding the mechanisms of *Plasmodium* sporozoites invasion at the molecular level as well as for the design of inhibitors specifically targeting the CD81-dependent entry step.

## Results

### Critical role of CD81 LEL in *P. yoelii* sporozoite infection

**Figure 1:**
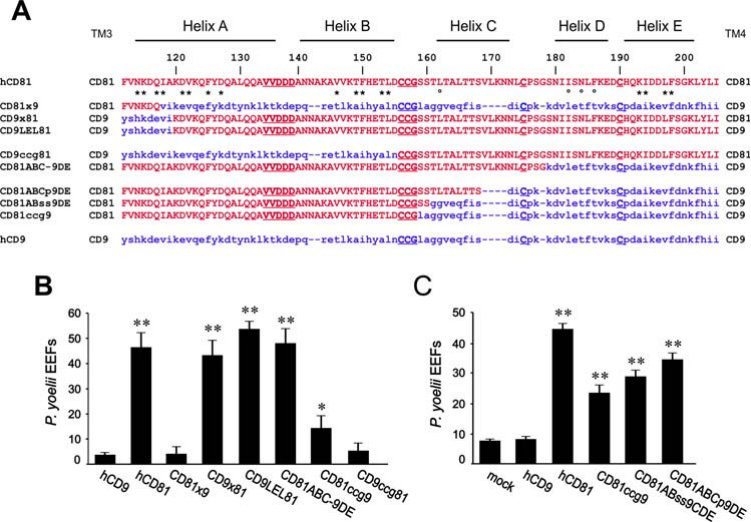
The A and B helices of CD81 LEL confer CD9/CD81 chimeric molecules the ability to support infection by *P. yoelii* sporozoites. A: Amino acid sequence alignment of CD81, CD9, and chimeras. Only the sequence of the LEL is shown. The origin of the flanking domains (TM3 and TM4) is shown on both sides of the sequence. The position of CD81 helices is indicated on the top of the alignment. CD81 residues are shown in red capital letters and CD9 residues in blue small letters. The CCG consensus site and other conserved cysteines, as well as a functionally important site (VVDDD) are underlined. CD81 LEL residues presumably in contact with the SEL are indicated with an asterisk. Open circles shows residues known to be involved in the interaction with HCV E2 glycoprotein. B and C: HepG2-A16 cells were transiently transfected with plasmids expressing CD9, CD81, or CD81/CD9 chimeras and infected two days later with *P. yoelii* sporozoites. After two days incubation, the number of EEF-infected cells was determined by immunofluorescence in triplicate wells. Results are expressed as mean±s.d. **, p<0.01 and *, p<0.05 as compared to CD9-transfected cells.

HepG2-A16 cells express very little CD81 at their surface and are not permissive to *P. yoelii* invasion. The few EEF observed were small and intranuclear, resulting from CD81-independent sporozoite entry through disruption of the cell plasma membrane [12]. We have recently shown that stable expression of human CD81 is sufficient to allow *P. yoelii* sporozoite entry and further differentiation into replicative exo-erythrocytic forms (EEF) [13],[14]. As shown in Fig. 1, transient expression of CD81, but not of CD9, rendered HepG2-A16 cells susceptible to *P. yoelii* infection. This differential ability of CD81 and CD9 to support infection opened the way to investigating CD81 domains important for infection using CD9/CD81 chimeras. Two previously characterized chimeras were first tested [35]. CD9×81 consists of the first three transmembrane domains of CD9 joined to the second half of CD81, comprising most of its large extracellular domain (excluding the very first residues) and the fourth transmembrane region (Fig 1A). This chimera rendered HepG2-A16 cells susceptible to *P. yoelii* infection while the reciprocal construction CD81×9 did not (Fig. 1B). This second chimera is known to be functional since it potentiated the toxicity of the diphtheria toxin to the same extent as CD9 [35]. Furthermore, a construct where CD9 LEL was exchanged with CD81 LEL (CD9LEL81) (Fig. 1A) was also able to support infection to the same extent as CD81 (Fig. 1B), pointing out the importance of CD81 LEL.

### The ability of CD81 to support *P. yoelii* sporozoite infection depends on the first half of CD81 LEL comprising the A and B helices

As swaps inside tetraspanin LEL abrogate the reactivity of most mAbs [25] (and table I), all subsequent chimeric CD9/CD81 molecules and CD81 mutants were fused to EGFP to facilitate the determination of expression level. Both CD81 and CD9LEL81 rendered HepG2-A16 cells permissive for infection when EGFP was fused to their C-terminus (Fig 1C and data not shown), indicating that the fusion with EGFP does not alter the function of the molecules with respect to *P. yoelii* infection.

**Table 1:** Recognition of CD81/CD9 chimeras by CD9 and CD81 mAbs.

		Construct									
Staining	mAb	CD81	CD9	CD9LEL81	CD9ccg81	CD81ccg9	CD9A-81BCDE	CD9A'-81BCDE	CD81ABC-9DE	CD9[81A]	CD9[81B]
**EGFP**		65	90	79	82	65	92	73	113	92	53
**CD81 mAb**	**1D6**	467	-	513	252	-	367	322	-	-	-
	**JS81**	539	-	459	-	-	-	-	51	-	-
	**JS64**	537	-	589	18	-	23	15	-	-	-
**CD9 mAb**	**10B1**	-	167	-	-	193	-	-	11	179	121
	**ALB-6**	-	552	-	-	64	-	-	-	178	-

The different chimeras in fusion with EGFP were transfected in Hepa1-6 cells, stained with the indicated mAbs and analyzed by flow-cytometry. The values correspond to the mean fluorescence intensity in the FL1 channel (EGFP) or the FL2 channel, after subtraction of the value obtained with cells labelled only with the secondary reagent. Other mAbs tested did not stain cells expressing any of the chimeras with swaps inside the LEL. These mAbs were TS81, 5A6, Z81, M38 (CD81); SYB-1, TS9, TS9b (CD9). (-): no staining.

**Figure 2:**
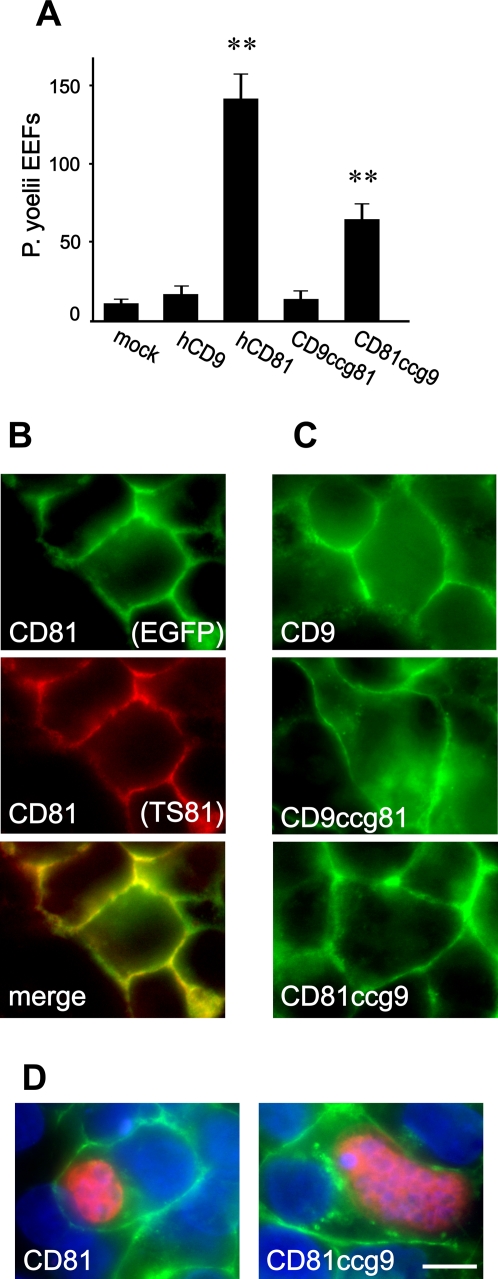
Reduced infection but normal schizont development in HepG2-A16 cells stably expressing CD81ccg9 chimera. A: HepG2-A16 cells stably expressing CD81, CD9, CD81ccg9 or CD9ccg81 (fused to EGFP) were infected with *P. yoelii* sporozoites. After two days incubation, the number of EEF-infected cells was determined by immunofluorescence in triplicate wells. Results are expressed as mean±s.d. **, p<0.01 as compared to mock-transfected cells. B: The localization of CD81 (fused to EGFP) stably expressed in HepG2-A16 cells was observed by fluorescence microscopy. Both the EGFP signal (green) and the labeling with the CD81 mAb TS81 (red) are shown. A merge image of the EGFP fluorescence and the mAb labeling is also shown (bottom). C: Localization of CD9, CD81ccg9 or CD9ccg81 (fused to EGFP) stably expressed in HepG2-A16. The images show the EGFP signal. D: HepG2-A16 cells stably expressing CD81-EGFP (left) or CD81ccg9-EGFP (right) were subjected to *P. yoelii* sporozoites infection. After 2 days incubation, the cells were fixed and labeled. The images are the merge of 3 different acquisitions: the green labeling corresponds to the EGFP signal. The red labeling corresponds to the labeling with anti-*Plasmodium* HSP70 antibodies and the blue labeling to DAPI fluorescence. Bar = 10 µm.

A molecule in which a swap between CD9 and CD81 was at the CCG consensus site (CD9ccg81; the CCG motif immediately follows the B helix, Fig. 1A) did not render HepG2-A16 cells susceptible to infection (Fig. 1B), suggesting an important role of the region comprising the A and B helices. Indeed, the reciprocal chimera CD81ccg9 supported *P. yoelii* sporozoite infection after transient transfection in HepG2-A16 cells (Fig. 1B–C), showing that CD81 A and B helices in a CD9 backbone are sufficient to confer HepG2-A16 cells the ability to support infection by *P. yoelii* sporozoites. These results were obtained both with EGFP-tagged constructs (Fig. 2) and non-tagged molecules (data not shown).

After transient transfection, the number of infected HepG2-A16 cells was significantly higher with CD81ccg9 than with CD9 (Fig. 1B–C), but usually remained very low. It was possible that the ability of this chimera to support infection *P. yoelii* was underestimated as a consequence of the low transfection efficiency of these cells (∼25%). Thus, to strengthen the conclusion that the CD81 A and B helices in a CD9 backbone are sufficient to render HepG2-A16 cells susceptible to infection, CD81ccg9 and CD9ccg81 chimeras were stably expressed in HepG2-A16 cells and compared to cells stably expressing CD81. As shown in Fig. 2A, cells expressing CD9ccg81 were not infected by *P. yoelii* sporozoites, in contrast to cells expressing CD81ccg9. The infection rate of cells expressing CD81ccg9 was about half that of CD81-expressing cells, despite a higher level of expression as determined by flow-cytometry (data not shown). Fluorescence microscopy showed that the cellular distribution of CD81ccg9 and CD9ccg81 was indistinguishable from that of CD81 (Fig. 2B, 2C). In addition, the parasite development was very similar in both cell lines. (Fig. 2D).

The fact that the level of infection obtained after transfection of CD81ccg9 was repeatedly lower than after CD81 transfection, suggested that in addition to the A-B region, the second half of CD81 LEL (comprising the C, D and E helices) may contribute to CD81 function during *Plasmodium* infection. A chimera in which a CD81/CD9 sequence switch was made just before the beginning of the D helix (CD81ABC-9DE) (Fig. 1A) was as efficient as CD81 in supporting infection by *P. yoelii* sporozoites when expressed in HepG2-A16 cells (Fig. 1B). This result indicates that CD81 D and E helices do not play a specific role, and points to a possible role of the C region. Two additional chimeras which restore 2 and 10 CD81 residues in the C region were constructed (CD81AB_ss_9CDE and CD81ABC_p_9DE). The level of infection in HepG2-A16 cells expressing these constructs was intermediate between the levels obtained after expression of CD81 and CD81ccg9, respectively (Fig. 1C).

### Residues at CD81 A–B junction are critical for *P. yoelii* infection

**Figure 3:**
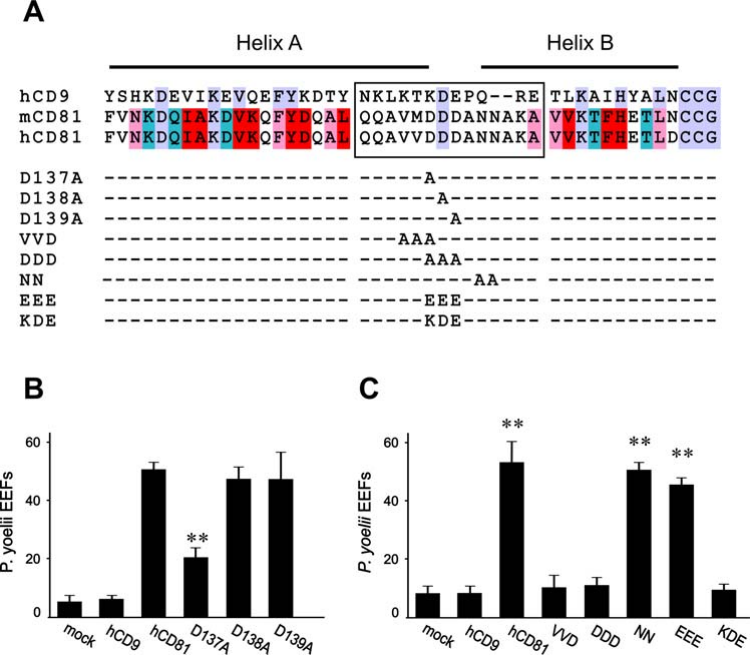
Mutations of residues at the A-B helix junction impair the ability of CD81 to support infection by *P. yoelii* sporozoites. A: Alignment of human CD81, mouse CD81 and human CD9. Residues that contribute to the stability of the subunit fold as reported by Kitadokoro et al. [30] are boxed in red. Additional residues that are buried according to the modelling of Seigneuret et al. [31] are boxed in pink. Some of the buried residues are also involved in the stabilization of the molecule and are therefore boxed in red. Residues potentially in contact with the small extracellular domain, according to the modelling of Seigneuret [36] and not fitting in the previous categories, are in blue. Finally, additional residues identical in both CD9 and CD81 sequences are in parma. The different mutants are also indicated on this alignment. The multiple mutants are designed as follow: VVD: VVD (135–137)→AAA; DDD: DDD (137–139)→AAA; NN: NN (141–142)→AA; EEE: DDD (137–139)→EEE; KDE: DDD (137–139)→KDE B and C: HepG2-A16 cells were transfected with the indicated mutants 48 hours before infection with *P. yoelii* sporozoites. After two days incubation, the number of EEF-infected cells in triplicate wells was determined by immunofluorescence. Results are expressed as mean±s.d. B: single mutations. **, p<0.01 as compared to cells transfected with WT CD81. C: triple or double mutations. **, p<0.01 as compared to mock-transfected cells

The above data indicated that in a CD9 backbone, the A and B helices of CD81 were necessary and sufficient to confer susceptibility to *P. yoelii* infection. We used the crystal structure of CD81 as well as published molecular modelling of tetraspanins to guide site-directed mutagenesis [30],[31],[36]. We reasoned that if CD81 plays a role as a receptor for a parasite protein, or as a partner molecule for such receptor, residues critically involved in these interactions would be solvent-exposed. We therefore excluded from mutagenesis analysis residues that contribute to the stability of the domain fold [30] or are buried in the molecule [31]. Recently, Seigneuret proposed that the hydrophobic face of CD81 LEL is in contact with the small extracellular loop [36], we therefore also excluded residues predicted to be in contact with the small domain from further analysis. Finally, since CD9 does not support *Plasmodium* sporozoite infection, residues common to both CD9 and CD81 were also not considered. From this analysis, we uncovered a stretch of 14 amino acids located around CD81 A–B helix junction, that are solvent-exposed and strongly different in CD9 (Fig. 3A). Notably, the loop corresponding to the A–B junction is highly acidic in CD81 with a row of 3 aspartic acids.

Each of the aspartate residues at A–B helix junction was mutated to Alanine (Fig. 3B). Mutation of D137 reduced by more than 60% the level of *P. yoelii* infection of transfected HepG2-A16 cells while the mutation of the two other D residues did not reduce infection (Fig. 3B). These data suggested that D137 could be part of a required site necessary for CD81 function during *Plasmodium* infection. To test this, 2 triple mutants overlapping the D137 residue, VVD (135–137)→AAA and DDD (137–139)→AAA, were generated (Fig. 3C). These mutations completely abolished the ability of CD81 to support *P. yoelii* infection in HepG2-A16 cells. In contrast, the mutations NN (141–142)→AA (Fig. 3C) or AV (145–146)→ET (not shown) had no effect on infection. All mutants were recognized by most CD81 mAbs tested (see below) and were expressed at cell surface to the same extent as WT CD81 in HepG2-A16 cells (data not shown) or in Hepa 1.6 cells (see below). Replacement of all 3 aspartic acids by glutamic acids (DDD (137–139)→EEE) did not change the level of infection indicating some tolerance in the length of the side chain provided that the global charge was respected (Fig. 3C). The importance of CD81 D137 is further confirmed by a mutant where CD81 DDD (137–139) residues were replaced by the corresponding amino acids of CD9 (CD81KDE) in CD9LEL81. Because substitution of all D by E did not affect the function of CD81, the main change in this mutant was the replacement of D137 by a lysine. As shown in Fig. 3C, this mutant was completely non-functional with respect to *P. yoelii* infection.

### Hepatocytic cell permissiveness to *Plasmodium* infection is conveyed by a short 21 residue CD81 region

**Figure 4:**
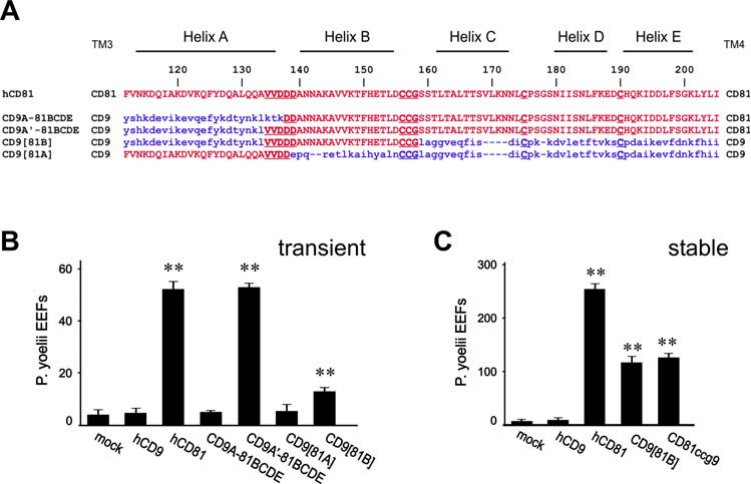
21 residues of CD81 in a CD9 backbone are sufficient to render hepatocytic cells susceptible to *P. yoelii* sporozoites infection. A: Amino acid sequence alignment of CD9, CD81 and chimeras. Only the sequence of the large extracellular loop of the different chimeras is shown. The origin of the flanking domains (TM3 and TM4) is shown on both sides of the sequence. The position of CD81 helices are indicated on the top of the alignment. CD81 residues are shown in red capital letters and CD9 residues in blue small letters. The CCG consensus site and other conserved cysteines, as well as a functionally important site (VVDDD) are underlined B: HepG2-A16 cells were transiently transfected with plasmids expressing CD9, CD81, or CD81/CD9 chimeras and infected two days later with *P. yoelii* sporozoites. After two days incubation, the number of EEF-infected cells in triplicate wells was determined by immunofluorescence. Results are expressed as mean±s.d. **, p<0.01 as compared to mock-transfected cells. C: HepG2-A16 cells stably expressing CD81, CD9, CD81ccg9 or CD9[81B] were infected with *P. yoelii* sporozoites. After two days incubation, the number of EEF-infected cells was determined in triplicate wells by immunofluorescence. Results are expressed as mean±s.d. **, p<0.01 as compared to mock-transfected cells.

Additional chimeras were generated to determine whether other CD81 residues in either the A or the B helix contribute to the ability of CD81 to support infection by *P. yoelii* sporozoites (Fig. 4). In these chimeras, swaps between CD9 and CD81 were made nearby the A–B junction (Fig. 4A). A CD9/CD81 chimera (CD9A-81BCDE) where the sequence switch was made immediately before D138 was not functional (Fig. 4B). This was expected since in this chimera D137 was replaced by the corresponding K residue in CD9. The chimera in which the sequence switch was made 3 residues before the predicted end of the A helix (before the VVD sequence in CD81: CD9A'-81BCDE) was on the contrary completely functional (Fig. 4B). Altogether these results confirm the importance of CD81 D137 residue and indicate that except for the last 3 residues, the A helix of CD81 can be replaced by that of CD9 without altering the ability to support *P. yoelii* infection.

A chimera in which CD9 A helix was replaced by that of CD81 (CD9[81A]) was not functional (Fig. 4B). In this chimera, the five CD81 residues (VVDDD (135–139)) the contribution of which has been demonstrated by site-directed mutagenesis (see above) are conserved (Fig. 4A), except D139 which is replaced by a functionally equivalent glutamic acid. This result, together with the fact that the chimera CD81ccg9 is partly functional, suggested that in addition to these 5 amino acids, other residues, located in the B region, contributed to the ability of CD81 to support *Plasmodium* infection of hepatocytic cells. Indeed, a chimera in which the end of the A helix and the B helix of CD9 were substituted by the 21 corresponding residues of CD81 (CD9[81B]: VVDDDANNAKAVVKTFHETLD) could support infection by *P. yoelii* sporozoites to the same extent as CD81ccg9 (Fig. 4B). Again, because the transfection efficiency in HepG2-A16 cells is low, the ability of CD9[81B] to support infection was more evident in HepG2-A16 cells stably expressing this chimera (Fig. 4C).

### The same CD81 determinants are required in a murine model

**Figure 5:**
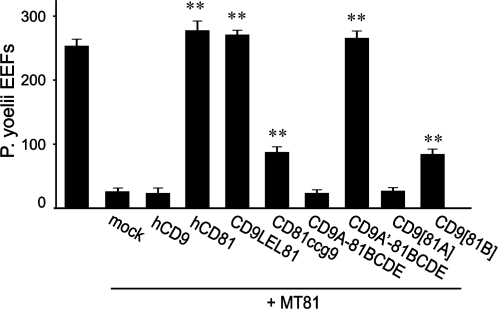
The same determinants are involved in a murine model. Hepa1-6 cells were transfected with the indicated plasmids 24 hours before infection with *P. yoelii* sporozoites in the presence of the anti-mouse CD81 mAb MT81 as indicated. The number of EEF-infected cells (mean±s.d.) was determined as described in the Materials and Methods section. **, p<0.01 as compared to MT81-treated, mock-transfected cells.

To check that the above results were not HepG2-A16 cell specific, we tested whether CD81 structural requirements were the same in the murine cell line Hepa1-6. This cell line expresses mCD81 and is permissive to *P. yoelii* infection. Treatment with an anti mouse CD81 mAb (MT81) prevents infection by this parasite [13] (Fig. 5). When cells are transfected with hCD81, they remain susceptible to infection despite MT81 treatment because the anti-mouse CD81 mAb does not recognize hCD81 (which can functionally replace mCD81 in this model [13]) (Fig. 5). These cells therefore represent an alternative model to study the molecular determinants of CD81 that play a role in the infection of hepatocytic cells by *P. yoelii* sporozoites.

As shown in Fig. 5, the chimeras CD9LEL81 and CD9A'-81BCDE that fully supported *P. yoelii* infection in HepG2-A16 cells restored the level of infection of MT81-treated Hepa1.6 cells to the initial level, as did hCD81. As in HepG2-A16 cells, CD81ccg9 and CD9[81B] partially restored infection. In contrast, hCD9, CD9A-81BCDE and CD9[81A] did not restore infection. In addition, The mutants VVD (135–137)→AAA and DDD (137–139)→AAA were unable to restore infection in MT81-treated Hepa1.6 cells (data not shown). Altogether, these data confirm the role of the 21-residues region comprising the A–B junction and the B helix.

### Expression of chimeras at cell surface and recognition by CD9 and CD81 mAbs

The EGFP signal obtained with the different constructs was reasonably similar after transfection in Hepa1.6 (table I) or HepG2-A16 cells (data not shown). The recognition of the different chimeras by a panel of 7 CD81 mAbs and 5 CD9 mAbs was tested after transfection in the murine hepatoma cell line Hepa 1.6 because the level of transfection is higher in this cell line that in HepG2-A16 cells (and thus the observed variations are more reliable), and because the high level of human CD9 in HepG2-A16 cells precluded the analysis with CD9 mAb.

The localization at cell surface of all constructs tested but one (CD81ABC-9DE) could be verified by labelling with certain CD9 or CD81 mAbs and flow-cytometry analysis. So far, all tetraspanin mAbs were shown to recognize the LEL [15]. Accordingly, CD9LEL81 was recognized by all CD81 mAb tested here (TS81, 5A6, Z81, M38, JS64, JS81, 1D6: table I). CD9×81 and CD81×9 have previously been shown to be recognized by CD81 and CD9 mAbs respectively [35].

Most mAbs tested here (TS81, 5A6, Z81, and M38 to CD81; SYB-1, TS9 and TS9b to CD9) failed to recognize chimeras with swaps inside the LEL (although in CD81LEL9, CD81×9 and CD9×81 the exchange between CD9 and CD81 is engineered a few residues after the predicted beginning of the LEL, these constructs are not considered here as molecules with a swap inside the LEL) (data not shown). The CD81 mAb JS64 and JS81, as well as the CD9 mAb ALB-6 could stain cells expressing certain chimeras, but at a much reduced level as compared to cells expressing WT CD81 or CD9 (table I). The mAb 1D6 is unique for its ability to recognize in ELISA assay a peptide corresponding to CD81 amino acids 179–193, encompassing the D helix and a few adjacent residues [33]. Accordingly, this mAb was found to recognize all constructs conserving CD81 D helix including CD9ccg81 and CD9A-81BCDE (Table I). Using 1D6, we could demonstrate that these two chimeras were expressed at a level similar to that of CD81 in transfected HepG2-A16 cells (data not shown), indicating that the lack of infection after transfection of these chimeras is not due to diminished surface expression. Additionally, 10B1 was described as the only CD9 mAb that recognizes a CD82/CD9 chimera in which the swap was done at the CCG consensus sequence [25]. This is consistent with its ability to recognize CD81ccg9, CD9[81A] and CD9[81B] (Table I). Importantly, the staining of cells expressing the functional chimera CD9[81B] is lower than the staining of cells expressing the non-functional chimeras CD81ccg9 and CD9[81A] (Table I). Thus the lack of activity of CD81ccg9 and CD9[81A] is not due to a lack of surface expression.

### A CD81 mAb recognizing the A–B junction does not block infection by *P. yoelii* sporozoites

**Figure 6:**
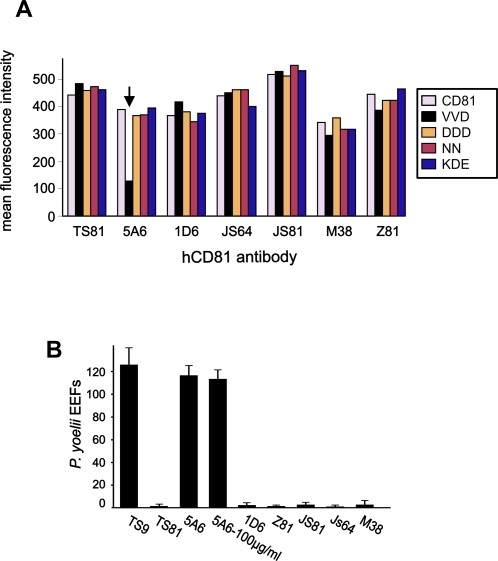
A CD81 mAb binds poorly to the non-functional mutant VVD (135–137)→AAA but does not block infection. A: Hepa 1–6 cells were transfected with the indicated construct in pEGFP-N3 and analyzed for the surface expression and recognition of the transgene by several CD81 mAb using flow-cytometry analysis. Data are expressed as mean fluorescence intensity. In this experiment, the antibodies were used at 20 µg/ml (JS64, M38, JS81) or at 1/100 ascitic fluid dilution (all other mAbs). B: HepG2-A16/CD81 cells were infected with *P. yoelii* sporozoites in the presence of the indicated mAbs at 25 µg/ml except when otherwise indicated. All mAbs are directed to CD81 except TS9 which is a CD9 mAb and does not inhibit *P. yoelii* infection.

The surface expression and the conformation of the CD81 mutants unable to support infection by *P. yoelii* sporozoites were tested. Hepa1.6 cells were transiently transfected with the different constructs and surface expression was assessed by flow-cytometry, using a panel of 7 CD81 mAbs produced in the mouse (Fig. 6A). Most anti-tetraspanin mAbs if not all do not recognize the denatured (reduced) protein indicating the recognition of conformational epitopes. This is confirmed by our analysis of chimeric CD9/CD81 molecules with swaps in the LEL, which were not recognized (or only at a low level) by all CD9 or CD81 mAbs except by mAb 10B1 to CD9 and 1D6 to CD81, both able to recognize the CDE region of the corresponding tetraspanin (Table I). All mutants were recognized by 6 CD81 mAbs with a staining very similar to the staining of WT CD81, therefore indicating a correct conformation. Interestingly, 5A6 had a ∼70% reduction of binding to the VVD (135–137)→AAA mutant. The fact that 5A6 was the only CD81 mAb to have a reduced ability to bind to this mutant indicates that the epitope recognized by this mAb critically depends on residues located at the A–B junction. This is coherent with the second Val being substituted by a Met in mouse CD81, which is not recognized by the mAb (Fig. 3A).

We then tested whether 5A6 could inhibit the infection of HepG2-A16 cells stably expressing CD81 by *P. yoelii* sporozoites. All CD81 mAbs tested here almost completely inhibited infection at 25 µg/ml, except 5A6 which had no effect on infection at concentrations up to 100 µg/ml (Fig. 6B). This is not due to a lower affinity of 5A6 mAb (data nor shown).

### The CD81 VVD (135–137)→AAA and DDD (137–139)→AAA mutants associate with CD9P-1 and EWI-2

**Figure 7:**
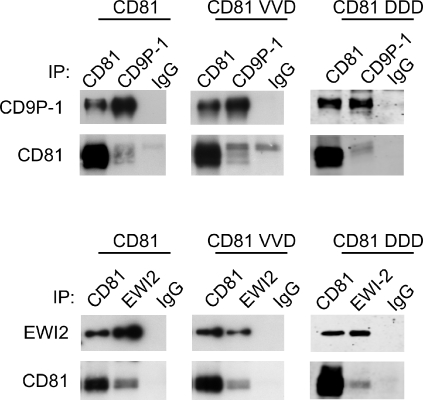
The VVD (135–137)→AAA and DDD (137–139)→AAA mutants unable to support infection by *P. yoelii* sporozoites interact with CD9P-1 and EWI-2. CHO cells were transiently transfected with WT or mutant CD81 plasmids (in pEGFP-N3), together with a CD9P-1 (top) or a EWI-2 (bottom) cDNA. After 48 h, the cells were lysed with digitonin and immunoprecipitations with antibodies against CD81, CD9P-1 and EWI-2 were performed. After electrophoresis and transfer, the membranes were incubated with biotin-labelled mAbs to CD81 (TS81), CD9P-1 (1F11) and EWI-2 (8A12). The mutants are designed as follows: VVD: VVD (135–137)→AAA; DDD: DDD (137–139)→AAA

The two major CD81 partners identified so far in hepatocytic cells are CD9P-1 and EWI-2, two Ig domain molecules [25]. Because tetraspanins have been shown in some cases to modulate the function of the proteins to which they associate, it was important to check whether the mutants unable to support infection by *P. yoelii* sporozoites still associate with these molecules. CHO cells were transfected with CD9P-1 or EWI-2 and either WT CD81, the VVD (135–137)→AAA mutant or the DDD(137–139)→AAA mutant (all in fusion with EGFP), and their interactions was tested by immunoprecipitation after cell lysis with digitonin (a detergent suitable to visualize direct interactions within the tetraspanin web). In preliminary experiments TS81 was shown to recognize the endogenous hamster CD81 molecule expressed by CHO cells, while 1D6 did not, so 1D6 was used for further analysis. As shown in fig 7, CD9P-1 and EWI-2 were co-immunoprecipitated with WT CD81 and the two CD81 mutants, and reciprocally the CD81 mutants were co-immunoprecipitated with CD9P-1 and EWI-2. This shows that the inability of the two mutants to support infection by *P. yoelii* sporozoites is not due to a loss of interaction with these partner molecules and further indicates that these mutants have a correct conformation.

### Mutants of the SEL β-sheet are not functional

**Figure 8:**
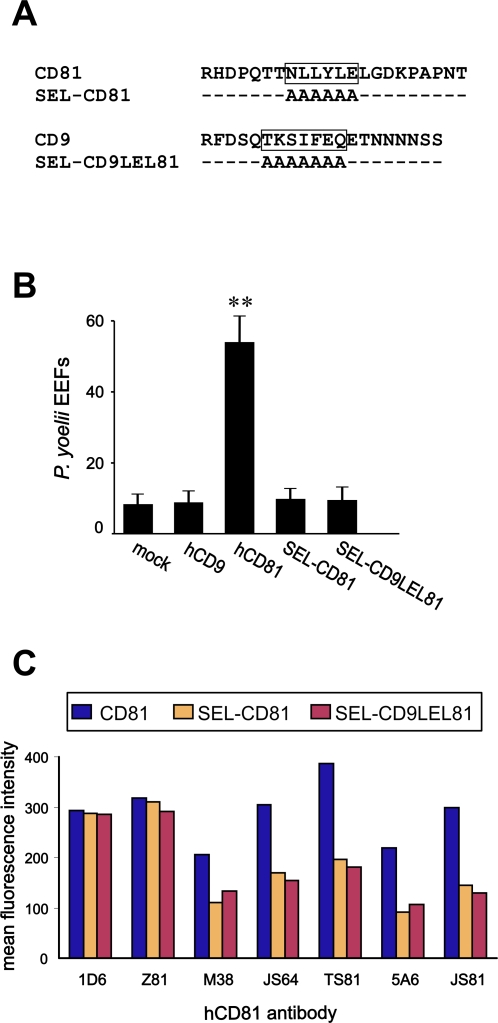
Mutation of the SEL β-sheet abolishes *P. yoelii* infection and alters the LEL conformation. A: Sequence of CD81 and CD9 SEL, and description of the SEL mutants. The boxes indicate the β-sheet [36]. B: HepG2-A16 cells were transiently transfected with plasmids expressing CD9, CD81, or CD81 molecules with mutations in the SEL, and infected two days later with *P. yoelii* sporozoites. After two days incubation, the number of EEF-infected cells was determined by immunofluorescence in triplicate wells. Results are expressed as mean±s.d. **, p<0.01 as compared to mock-transfected cells. C: Hepa 1–6 cells were transfected with the indicated constructs and analyzed for the surface expression of the transgene by flow-cytometry analysis. Data are expressed as mean fluorescence intensity. In this experiment, the antibodies were used at 20 µg/ml (JS64, M38, JS81) or at 1/100 ascitic fluid dilution (all other mAbs).

Our initial data, using CD9×81 and CD9LEL81 chimeras, indicated that CD81 SEL could be replaced with that of CD9 without affecting the ability to support *P. yoelii* sporozoites infection (Fig. 1). Both CD9 and CD81 SEL are predicted to contain a β-sheet that was proposed to tightly interact with the LEL [36]. To further investigate the role of the SEL, residues of this β-sheet were mutated to Ala. Two mutants were designed: SEL-CD81, in which NLLYLE (43–48) was mutated to AAAAAA in the hCD81 molecule, and SEL-CD9LEL81, in which TKSIFEQ (41–47) was mutated to AAAAAAA in the CD9LEL81 chimeric molecule (Fig. 8A). None was able to confer cell susceptibility to *P. yoelii* infection (Fig. 8B). These mutants are normally expressed at the cell surface as shown by the binding of the non-conformational mAb 1D6 (Fig. 8C). Five out of 7 CD81 mAb tested showed a reduced binding to cells expressing this mutant as compared to cells expressing WT CD81, indicating that the conformation of the LEL of these mutants is modified (Fig. 8C).

## Discussion

The molecular mechanisms underlying *Plasmodium* sporozoite invasion of hepatocytes remain poorly characterized [1]–[4]. So far, only one host molecule, the tetraspanin CD81, has been shown to play a crucial role in the infection by several *Plasmodium* species, including *P. falciparum*, the deadliest human parasite [11]–[14]. In this report, we have investigated CD81 domains or residues that are critical for the ability of CD81 to confer hepatocyte permissiveness to *Plasmodium* sporozoites. We have delineated a stretch of 21 amino acids, starting from the end of the A helix to the end of the B helix, that are sufficient, when present in a CD9 backbone, to render hepatocytic cells permissive to infection. Site-directed mutagenesis in this region has highlighted the crucial role of D137 and adjacent residues. This is the first demonstration that this region of CD81 (and other tetraspanins) is functionally important in a physiological or pathological process.

### CD81 LEL domains and residues required for hepatocyte permissiveness to *Plasmodium* infection

**Figure 9:**
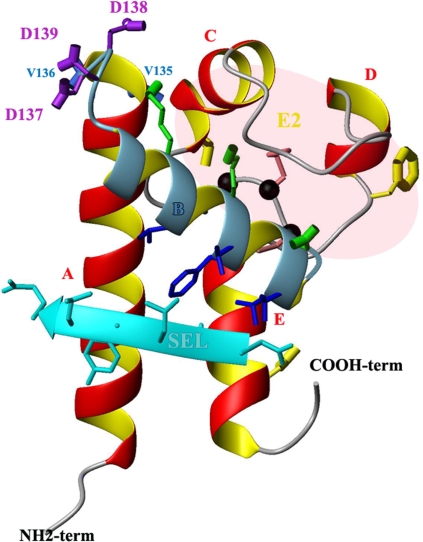
3D structure of CD81 LEL. The drawing of CD81 LEL (PDB #1g8q) was generated in MolMol. Four helices (A, C, D, E) are drawn in red while the B helix, crucial for *P. yoelii* infection is displayed in blue. The black balls indicate the CCG ubiquitous motif. The crucial D137 as well as D138 and D139 are in purple while V135 and V136 are in royal blue. Residues V146, T149, F150, T153 and L154 putatively involved in contact with the SEL are indicated in dark blue. T163, F186 and D196 residues, in yellow, have been reported to play a role in the HCV E2 glycoprotein binding to CD81-LEL. Residues V135, V136, T163, F186 and D196 projected backward, behind the drawing plane. The two disulfides bridges are colored light coral. Hydrophilic residues K144, K148 and E152 located on the top of the B helix are in green. The SEL, in cyan, is in front of the drawing plane.

So far specific tetraspanin functions have been linked to residues in the LEL. Only few studies have investigated whether the A–B region contributes to tetraspanin specific functions. This region was found not to directly contribute to the interaction of CD9 with EWI-2 or CD9P-1 ([25]; S. Charrin and E. Rubinstein, unpublished data) and replacement of most of this region did not prevent the interaction of CD151 with the integrin α3β1 [37]. In contrast, our results indicate that the A–B region of CD81 on a CD9 backbone is sufficient to allow infection of hepatocytic cells by *P. yoelii* sporozoites. More specifically, a major part of the A helix can be replaced by the corresponding region of CD9 without altering sporozoite infection, and substitution of 21 amino acids of CD9 consisting of the end of the A helix and the B helix with the corresponding residues of CD81 yielded a chimera that supports to some extent infection by *P. yoelii* sporozoites. The key role of some residues was demonstrated by site-directed mutagenesis. Importantly, these residues were first selected after an analysis of CD81 3D structure [30],[31],[36], looking for residues that were solvent-exposed (Fig. 3A, Fig. 9). Among these solvent-exposed residues, D137 plays a key role since its mutation together with adjacent residues completely abolished the ability of CD81 to support *P. yoelii* infection, despite normal surface expression and conformation as determined by the recognition by several conformation-dependent mAbs and the ability to interact with CD9P-1 and EWI-2.

We have demonstrated that a chimeric molecule in which a swap between CD81 and CD9 was done immediately before D helix (CD81ABC-9DE) was completely functional with respect to *Plasmodium* sporozoite infection (Fig. 1). The D regions of CD9 and CD81 are completely different both in structure and amino acid sequence [15],[31], excluding the possibility that the D region of CD9 could functionally replace that of CD81. Thus the D region of CD81 does not play a direct role in the entry of *Plasmodium* sporozoites. This is consistent with the fact that both human and murine CD81 molecules support *P. yoelii* sporozoite infection [13](and this study), despite the fact that the D region is the most divergent region of mouse and human CD81 molecules (only 59% conservation, 66% similarity). This observation contrasts with most previous studies, if not all, that have pointed to a critical role for residues in the D domain for specific tetraspanin functions. Thus, a triple mutation in the D region of CD9 abolishes its activity in gamete fusion and another one prevents CD151 interaction with the integrins α3β1 and α6β1, as well as the ability of these integrins to support cellular cable formation in matrigel [34],[38]. Two residues in the D domain are also essential for the ability of CD9 to upregulate the binding of diphtheria toxin to its receptor, the transmembrane precursor of Heparin-Binding EGF [39].

The CD9/CD81 chimeras containing CD81 B helix but in which the C helix was that of CD9 (CD81ccg9 and CD9[81B]) had a reduced ability to support infection by *P. yoelii* sporozoites as compared to WT CD81. This may suggest that this region of CD81, although not essential, contributes to the function of CD81 during infection. However, two CD81/CD9 chimeras that progressively restored CD81 residues in the C regions progressively restored the ability to support *P. yoelii* invasion (Fig 1C), suggesting an indirect role for this region. The folding of the LEL brings the C helix in proximity to the A–B junction and almost on the top of the B helix (see Fig. 9). The CD9 C helix is 4 amino acids shorter than that of CD81 and is predicted to form a β strand instead of a α helix [31]. It is therefore possible that the C region of CD9 folds in a way that hampers the interaction of key residues at the A–B junction or in the B helix with other proteins.

### Possible mechanisms through which CD81 may support *Plasmodium* infection

How CD81 allows entry of *Plasmodium* sporozoites into hepatocytic cells is still unknown. In one hypothesis, CD81 would serve as a receptor for a sporozoite protein. However, several data are not consistent with this model. First, a recombinant CD81 LEL fused with GST did not block infection of hepatocytes by sporozoites, and did not bind to sporozoites [11], while a similar protein inhibited HCV infection, probably by interacting with the HCV E2 envelope glycoprotein, a CD81 ligand [40]–[42]. Additionally, we have shown here that the loop separating the A and B helices of CD81 LEL is critical for the function of CD81 during *P. yoelii* sporozoite infection (Fig. 3). If CD81 was a receptor for a sporozoite protein, it would be expected that mAbs that require this region for the recognition of CD81 are the most potent at inhibiting infection. This is not the case since the only mAb (5A6) that requires this critical site for maximum binding does not block infection, in contrast to all other CD81 mAbs (Fig. 6).

Another hypothesis is that CD81 modulates the activity of a molecule to which it associates, that could function as a sporozoite receptor. This hypothesis is based on the ability of tetraspanins to interact with many other integral proteins, and to functionally modulate these proteins [15]–[17]. For example, the expression of CD9 increases by several orders of magnitude the ability of diphtheria toxin to bind to its receptor, the transmembrane precursor of Heparin-Binding EGF [35],[43]. In this hypothesis, not all CD81 molecules but only those that associate with this particular molecular partner would be functionally competent for infection. If CD81 A–B junction participates in the interaction with such a partner protein, mutations in this region may abolish the interaction of CD81 with this partner, and as a result may prevent *Plasmodium* sporozoite entry. Furthermore, this association could conceal the A–B region and therefore mAbs such as 5A6 that require this region for optimal binding would not recognize the CD81 molecules present in this CD81-partner complex. Because only this subset of CD81 molecules would play a role in the infection by *Plasmodium* sporozoites, this model explains why 5A6, which is the only mAb that requires CD81 A–B region for binding is also the only mAb that fails to inhibit *P. yoelii* and *P. falciparum* sporozoite infection. The identification of an anti-tetraspanin mAb that fails to recognize particular complexes is not unprecedented. Indeed, a CD151 mAb fails to recognize the fraction of CD151 molecules associated with integrins because its epitope overlaps the integrin-binding site [22],[38]. Thus, the inability of 5A6 to inhibit infection while interacting with a critical region of CD81 is consistent with the hypothesis that CD81 functions through the regulation of an associated protein.

If 5A6 fails to recognize a pool of CD81 associated with a particular partner molecule, one would expect to observe differences in the pattern of proteins co-immunoprecipitated with CD81 using 5A6 or other CD81 mAb. So far we have found that 5A6 and TS81 immunoprecipitates collected after cell lysis with digitonin, a detergent appropriate to visualize primary complexes within the tetraspanin web, were very similar (data not shown). However, this finding does not preclude the hypothesis that 5A6 fails to recognize a fraction of CD81 associated with a particular partner, because digitonin probably dissociates certain primary complexes [25]. The major known CD81 partners in hepatocytes are CD9P-1 and EWI-2, and the mutations DDD (137–139)→ AAA and VVD (135–137)→AAA that abrogate CD81 activity in sporozoite infection did not alter its capacity to associate with these two molecules. This finding strongly suggests that the interaction of CD81 with these molecules does not play a direct role in the infection by *Plasmodium* sporozoites. Efforts to identify additional CD81 partner molecules will provide insights into the mechanisms by which CD81 supports infection by *Plasmodium* sporozoites.

### CD81 SEL stabilizes the conformation of the large extracellular loop

The SEL of most tetraspanins has a conserved secondary structure, namely a small predicted β-strand flanked by non regular structure stretches [36]. We have mutated the residues of this β-strand to Ala in CD81 and in a chimera in which the CD9 LEL was exchanged with that of CD81 (CD9LEL81). Both mutants were expressed at cell surface similarly to WT CD81, as determined by the staining with a conformation-independent mAb, 1D6. This contrasts with a previous study in which a CD81 mutant with substitution of the SEL with a linker was partially retained intracellularly [44]. Thus, CD81 SEL β-sheet does not contribute to the transport of CD81 to the cell surface.

The two SEL mutants do not support *P. yoelii* sporozoite infection. It is unlikely that the SEL directly participates to the interaction with a parasite ligand or with a partner molecule since chimeras in which the SEL and the LEL are respectively that of CD9 and CD81 (CD9×81 and CD9LEL81) are functional, despite the fact that the sequences of CD9 and CD81 SEL are highly divergent. We have shown that the two SEL mutants have an altered conformation as shown by the reduction of binding of 5 CD81 mAbs out of 7 tested. These data suggest that the SEL helps stabilizing the LEL active conformation. They are consistent with molecular modelling predicting that the small hydrophobic SEL β-strand packs in a conserved hydrophobic groove of the LEL [36]. Most of the LEL residues that are in contact with the SEL in the modeled CD81 structure are either size or polarity-conserved, or both, in CD9 (M. Seigneuret, personal communication). Therefore, it is probable that the LEL groove of CD81 can accommodate the CD9-SEL (and vice versa).

### Comparison of CD81 mechanism of action in HCV and *Plasmodium* infection

CD81 is essential for the infection of hepatocytic cells not only by several *Plasmodium* species, but also by the hepatitis C virus [19]. The wide distribution of CD81 suggests that CD81 expression is not the sole determinant of HCV and *Plasmodium* sporozoite tissue tropism. The tight junction protein Claudin-1 was recently shown to be required for HCV entry and to contribute to the virus tropism [45], but it is not necessary for *P. yoelii* invasion (Yalaoui. S et al; unpublished data). CD81 LEL is critical for the ability of CD81 to confer susceptibility to HCV glycoprotein-mediated infection [46]. The mutation of residues that are different in the human protein and in CD81 proteins from uninfectable species, as well as random mutagenesis, pointed to the key role of residues in the C and D helices for the binding of CD81 to the HCV envelope glycoprotein E2 [33], [46]–[48]. A hydrophobic Phe residue present in the D helix is in particular essential for the interaction of CD81 with the HCV envelope glycoprotein E2 in vitro [33], [46]–[48], although the importance of this residue during HCV infection is controversial [46],[48]. We have in contrast excluded a role for the D region during *P. yoelii* infection and highlighted a role for the AB helix junction and the B helix. Interestingly, the B helix and the residues that have been implicated in E2 binding are on different sides of CD81 (Fig. 9). Our data indicate additional differences in the mechanisms by which CD81 supports *Plasmodium* and HCV infections. Indeed, the CD81 mAb 5A6 is efficient in blocking the binding of the HCV envelope protein E2 to CD81 [49] and HCV infection [41],[50],[51]. We therefore predict that if a CD81 partner interacting through the A–B junction is shown to play a key role during the infection by malaria sporozoites, it will not play a crucial role during HCV infection.

## Materials and Methods

### Antibodies

Several mAb used in this study were generated in our laboratory and were described elsewhere [13],[20],[21],[24],[25]. These include TS81 specific for hCD81; SYB-1, ALB-6, 10B1, TS9 and TS9b specific for hCD9; 1F11 and 8A12 that respectively recognize CD9P-1 and EWI-2; MT81 is a rat an anti-mouse CD81. 5A6 and 1D6, two anti-hCD81 were kindly provided by Dr. S. Levy [33]. M38 and Z81, two anti-hCD81, were a gift of Dr. O. Yoshie [52] and Dr. F. Lanza [53], respectively. JS64 was purchased from Beckman-Coulter (Marseille, France) and JS81 from BD biosciences (San Jose, CA).

### Construction of CD9/CD81 chimeric molecules and mutagenesis

The plasmids encoding CD81, CD9, CD9P-1 and EWI-2, as well as the chimeras CD9×81 and CD81×9 have been previously described [24],[25],[35]. Additional constructs were engineered by classical PCR protocols using overlapping oligonucleotides. The constructs were inserted into pcDNA3 (Invitrogen, Cergy Pontoise, France) and/or pEGFP-N3 (Clontech, Mountain view, CA) to make EGFP fusion proteins. The human CD81 was mutated using the QuickChangeTM site-directed mutagenesis kit from Stratagene (Amsterdam, The Netherlands), according to the manufacturer's specifications, except for the design of primers which was done according to the method of Zheng et al. [54].

### Cells, transfection and generation of stably transfected cell lines

The human hepatocarcinoma cell line HepG2-A16 [55], the mouse hepatoma cell line Hepa1-6 (ATCC CRL-1830) and CHO (Chinese Hamster Ovary) cells were cultured in DMEM (Invitrogen) supplemented with 10% FCS (Biowest, Nuaillé, France), 2 mM glutamine and antibiotics.

For infection, HepG2-A16 and Hepa1-6 cells were seeded in 8-chamber plastic Lab-Tek slides (10^5^ cells) (Nalge Nunc International, Cergy Pontoise, France) or in 96 wells microplate (2×10^4^ cells) 24 h hours before transfection. Cells were transfected using Lipofectamine 2000 (Invitrogen) according to the manufacturer's instructions and infections were done two days later. For generation of stable HepG2-A16-transfectants, pEGFP-N3 constructs were linearised by digestion with Afl III and EcoO109I restriction enzymes and cleaned up using the QIAquick PCR purification kit (Qiagen) before transfection. G418 (Gibco) was added to cells 2 days later at the concentration of 1 mg/ml. After a 3-week selection period, EGFP expressing cells were sorted using a FACS-VANTAGE-DIVA (Becton-Dickinson-BDIS). The cells expressing CD81, CD81ccg9, CD9ccg81 and CD9 were further cloned by limiting dilution.

### Parasites and invasion assay

*Plasmodium* infection into HepG2-A16 and MT81-treated Hepa1-6 were performed as described before [12],[14]. *P. yoelii* (265BY strain) sporozoites were obtained from dissection of infected *Anopheles stephensi* mosquito salivary glands. For infection, the cells were inoculated with 10^5^ *P. yoelii* sporozoites in 8 wells Lab-Tek culture chambers (Nalge Nunc International, Cergy Pontoise, France) or 2×10^4^ sporozoites in 96 wells culture plates. After 3 h at 37°C, cultures were washed and further incubated in fresh medium for 36 h before fixation and parasite labelling using an antibody again *Plasmodium* heat shock protein 70 (HSP70) [11]. Quantification of infected cells in triplicate wells was done either under fluorescence microscope (Lab-Tek) or by using the Odyssey Infrared Imaging System (LI-COR Biosciences) as described previously [56]. For antibody inhibition assays, the mAbs were added at a concentration of 25 µg/ml (unless otherwise specified) at the same time as the parasites. The results are expressed as mean number of EEF/well +/- SD. The data were analysed for statistical significance using the OneWay ANOVA followed by the Tukey multiple comparison test.

### Immunofluorescence analysis

For flow cytometric analysis, cells were detached using a non-enzymatic solution (Invitrogen), washed and stained with primary mAbs. After 3 washes in culture medium, cells were incubated with 10 µg/ml PE-labeled secondary antibody (Beckman Coulter) and washed again three times. All incubations were performed for 30 minutes at 4°C. Analysis of cell-surface staining was performed using a FACSscan flow cytometer (Becton-Dickinson, San Jose, CA, USA) using appropriate compensations.

### Immunoprecipitation and western blotting

CHO cells were electroporated as described previously [25]. The cells were lysed 48 h later in 30 mM Tris, pH 7.4, 150 mM NaCl, 0.02% NaN3, protease inhibitors and 1% digitonin (high purity; Calbiochem, San Diego, CA, USA). Immunoprecipitations were then performed as described previously [24],[25]. The composition of the different immunoprecipitates was analyzed by western-blotting using biotin-labelled mAbs.
